# Altered coupling of resting-state cerebral blood flow and functional connectivity in Meige syndrome

**DOI:** 10.3389/fnins.2023.1152161

**Published:** 2023-05-03

**Authors:** Aocai Yang, Bing Liu, Kuan Lv, Jixin Luan, Pianpian Hu, Hongwei Yu, Amir Shmuel, Shijun Li, Hong Tian, Guolin Ma, Bing Zhang

**Affiliations:** ^1^Department of Radiology, China-Japan Friendship Hospital, Beijing, China; ^2^Graduate School of Peking Union Medical College, Chinese Academy of Medical Sciences and Peking Union Medical College, Beijing, China; ^3^Peking University China-Japan Friendship School of Clinical Medicine, Beijing, China; ^4^McConnell Brain Imaging Centre, Montreal Neurological Institute, McGill University, Montreal, QC, Canada; ^5^Departments of Neurology and Neurosurgery, Physiology, and Biomedical Engineering, McGill University, Montreal, QC, Canada; ^6^Department of Radiology, First Medical Center of Chinese PLA General Hospital, Beijing, China; ^7^Department of Neurosurgery, China-Japan Friendship Hospital, Beijing, China; ^8^Department of Radiology, The Affiliated Drum Tower Hospital of Nanjing University Medical School, Beijing, China

**Keywords:** Meige syndrome, arterial spin labeling, cerebral blood flow, functional magnetic resonance imaging, functional connectivity strength

## Abstract

**Introduction:**

Meige syndrome (MS) is an adult-onset segmental dystonia disease, mainly manifested as blepharospasm and involuntary movement caused by dystonic dysfunction of the oromandibular muscles. The changes of brain activity, perfusion and neurovascular coupling in patients with Meige syndrome are hitherto unknown.

**Methods:**

Twenty-five MS patients and thirty age- and sex-matched healthy controls (HC) were prospectively recruited in this study. All the participants underwent resting-state arterial spin labeling and blood oxygen level-dependent examinations on a 3.0 T MR scanner. The measurement of neurovascular coupling was calculated using cerebral blood flow (CBF)-functional connectivity strength (FCS) correlations across the voxels of whole gray matter. Also, voxel-wised analyses of CBF, FCS, and CBF/FCS ratio images between MS and HC were conducted. Additionally, CBF and FCS values were compared between these two groups in selected motion-related brain regions.

**Results:**

MS patients showed increased whole gray matter CBF-FCS coupling relative to HC (*t* = 2.262, *p* = 0.028). In addition, MS patients showed significantly increased CBF value in middle frontal gyrus and bilateral precentral gyrus.

**Conclusion:**

The abnormal elevated neurovascular coupling of MS may indicate a compensated blood perfusion in motor-related brain regions and reorganized the balance between neuronal activity and brain blood supply. Our results provide a new insight into the neural mechanism underlying MS from the perspective of neurovascular coupling and cerebral perfusion.

## 1. Introduction

Meige syndrome (MS), also known as blepharospasm-oromandibular dystonia, was first described and named in 1910 by French neurologist Henry Meige (Juvarra et al., 1981). MS is an idiopathic segmental craniocervical dystonia disease. The age range of the patients is approximately about 30 to 70 years old (Sabesan, 2008). The prevalence rate in males is higher than females and the ratio of males to females is in the range of 1:2 to 1:3 (Pandey and Sharma, 2017). The course of the disease is varied, and eventually manifests as blepharospasm or with oromandibular dystonic motor symptoms (Zalyalova, 2015). To date, the pathogenesis and etiology of MS is not fully understood. Experts opinions are divided, with some suggesting that the basal ganglia is dysfunctional (Tanner et al., 1982), and others suggesting that the lack of inhibition at various levels in the central nervous system results in hyperactivity in patients (Prescott et al., 2013). Such abnormal changes in inhibitory ability can occur in the brain stem, spinal cord, basal ganglia, and cerebral cortex.

Using conventional head magnetic resonance imaging (MRI), it is difficult to detect abnormal brain changes in MS patients; however, advanced MRI technologies such as blood-oxygen-level dependent (BOLD) functional MRI (fMRI) and arterial spin labeling (ASL) MRI may provide important pathophysiological information for understanding the MS. Previous studies of fMRI found that MS patients exhibited altered functional connectivity (FC) at rest in widespread brain regions including basal ganglia, cerebellar, primary/secondary sensorimotor, and visual areas (Baker et al., 2003; Jochim et al., 2018). Cerebral blood flow (CBF) in the resting-state reflects a basic perfusion in the brain. It tightly correlates with the normal function, glucose metabolism and oxygen consumption of the brain, which is a crucially biomarker for indicating cerebral metabolic level. ASL is a non-invasive MR technique that can measure CBF by using endogenous water spins as the tracer without using an exogenous contrast agent (Su et al., 2017). In the normal state, spontaneous brain activity is the cause of the larger part of cerebral energy metabolism. Prior studies combined resting-state BOLD and ASL to explore the relationship between CBF and FC, and found that CBF is tightly associated with FC strength (FCS) in normal state (Liang et al., 2013). Changes in CBF-FCS coupling were found in healthy aging (Galiano et al., 2020). In addition, altered CBF-FCS coupling has been reported in several different diseases, such as generalized anxiety disorder, Wilson’s disease primary open angle glaucoma and schizophrenia (Zhu et al., 2017; Hu et al., 2019; Chen et al., 2021; Wang et al., 2021). According to the validated neurovascular coupling theory, hub regions with higher spontaneous brain activity and FCS are associated with greater demand for cerebral perfusion (Kuschinsky, 1991; Venkat et al., 2016). However, to date, using ASL technology to explore changes in CBF in patients with Meige syndrome has not been reported. Therefore, we hypothesized that neurovascular coupling may change in MS and can be indirectly detected by the correlation analysis of CBF-FCS.

Thus, in this study, we firstly investigated the CBF-FCS correlation coefficient across voxels, and conducted the voxel-wise analyses of CBF/FCS ratio, CBF, and FCS in MS patients and healthy control subjects (HC). Additionally, we evaluated the abnormalities of CBF and FCS in motor-related brain regions in MS. We explored a new neuroimaging biomarker of MS and provided some new insights and evidence into the pathogenesis of this disease.

## 2. Materials and methods

### 2.1. Subjects

The ethics committee of China-Japan Friendship Hospital has approved this study, and the signed informed consents were obtained from all participants.

Twenty-seven MS patients were prospectively recruited during their inpatient stay in the Department of Neurosurgery at China-Japan Friendship Hospital from September 2020 to January 2022. The inclusion criteria of MS patients were as follows: (a) All patients were diagnosed with primary Meige syndrome by one experienced senior neurosurgeon based on standard clinical criteria (Pakkenberg et al., 1987; Pandey and Sharma, 2017); (b) No abnormal brain changes in conventional MRI examination; (c) No treatment within the past 3 months; (d) Right-handed; and (e) The MRI images are complete and no artifacts. Exclusion criteria of MS patients were as follows: (a) Brain organic diseases; (b) Neuropsychiatric diseases; (c) Severe history of lung, heart, liver and kidney diseases; (d) MRI contraindications; and (e) Other dystonic disorders other than MS. Healthy controls (HC) were recruited from the local communities. The inclusion conditions of HC were as follows: (a) No psychoses and neurological diseases; (b) No abnormal brain lesions in conventional MRI examination; (c) Neurological tests were normal; (d) Right-handed; and (e) The MR images are complete and show no artifacts. All patients with MS underwent Burke-Fahn-Marsden dystonia rating scale-movement (BFMDRS-M) and blepharospasm disability index (BSDI) scale (Jankovic et al., 2009) to assess disease severity. All participants received conventional head MRI, resting-state BOLD and 3D-PCASL MR examinations. Two MS patients were excluded because of image artifacts. Twenty-five MS patients and thirty healthy controls were finally included in this study.

### 2.2. MRI data acquisition

Magnetic resonance examinations were conducted on a 3.0 T MR scanner (Discovery MR750 scanner; GE Medical Systems, USA) with eight-channel head coils. The scanning protocol included three dimensional pseudo-continuous ASL (3D-PCASL) sequence for perfusion imaging, single-shot gradient recalled echo-planar imaging (GRE-SS-EPI) sequence for resting-state BOLD images, three-dimensional fast spoiled gradient-echo (3D FSPGR) sequence for T1-weighted anatomical images. Acquisition parameters were as follows: (1) 3D-PCASL: repetition time (TR) = 4817 ms, echo time (TE) = 14.6 ms, slice thickness = 4 mm, post-label delay = 1525 ms, field of view (FOV) = 240 mm × 240 mm, bandwidth = 62.5 Hz/pixel; (2) GRE-SS-EPI: TR = 2000 ms, TE = 30 ms, flip angle = 90°, slice thickness = 3.5 mm, slice spacing = 0.7 mm, FOV = 224 mm × 224 mm, matrix = 64 × 64, NEX = 1, scanning time = 8 min, 240 time points; (3) 3D-FSPGR: TE = 2.9 ms, TR = 6.7 ms, flip angle = 12°, bandwidth = 31.25 Hz/pixel, slice thickness = 1.0 mm, FOV = 256 mm × 256 mm.

During the scanning, all subjects were requested to relax, close their eyes, and remain as still as possible, not to think about anything and not to fall asleep. All raw image data were visually examined to exclude subjects with visible image artifacts.

### 2.3. Data processing and analysis

#### 2.3.1. CBF processing

The CBF maps were calculated from the ASL data with Functools perfusion software in GE (General Electric Healthcare). In short, the CBF maps were derived from the ASL difference images and the proton density weighted reference images using a single compartment model after the subtraction of the label images from the control images (Buxton et al., 1998; Xu et al., 2010).

Individual CBF images in native space were directly co-registered to the perfusion template in SPM8^1^ and spatially normalized to Montreal Neurological Institute (MNI) space. Normalized CBF maps were resampled into isotropic voxel size of 3 mm × 3 mm × 3 mm and the non-brain tissue were removed. Then, CBF maps were further standardized into z-scores (zCBF). Finally, the standardized CBF maps were spatially smoothed with a 6 mm × 6 mm × 6 mm full-width at half maximum (FWHM) Gaussian kernel.

#### 2.3.2. fMRI data processing

The pre-processing of rs-fMRI data was conducted using a standard pipeline in DPABI (Yan et al., 2016). In short, the first ten volumes were removed, and then the remaining volumes were corrected for slice timing and head motion. Participants’ data with more than 3°rotation and/or 3 mm translation of head motion were excluded. Then, several nuisance covariates were regressed out, including 24 Friston of head motion parameters, polynomial trend, the average global signal, and signals averaged over the white matter and cerebrospinal fluid. The datasets were bandpass filtered within 0.01−0.08 Hz frequency range. Individual T1-weighted anatomical images were co-registered to the corresponding mean fMRI images, and then segmented and non-linearly normalized into MNI space. Finally, filtered fMRI images were also spatially normalized into the MNI space using the above mentioned transformations and resampled into 3-mm cubic voxels.

We computed the “degree centrality” of each voxel by calculating the Pearson’s correlation coefficients between the BOLD time courses of all pairs of voxels within the gray matter mask (Number of voxels = 67451). The threshold of positive FC was set to 0.2 in order to eliminate negative or false correlations from background noise (Liang et al., 2013; Zhu et al., 2017; Chen et al., 2021). The voxel-to-voxel FC lower than 0.2 was set to zero. The FCS maps were calculated by averaging the values of functional connectivity between a given voxel and all other voxels (Liang et al., 2013). Then, the FCS maps were standardized into z-scores (zFCS) and spatially smoothed with a 6-mm FWHM Gaussian kernel.

#### 2.3.3. CBF-FCS coupling analysis

The relationship between zCBF and zFCS was evaluated through the correlation analyses across voxels in each subject within the whole-cerebrum gray matter (Liang et al., 2013). According to prior studies (Liang et al., 2013; Zhu et al., 2017; Wang et al., 2021), the effective degree of freedom (*df*_*eff*_) of correlation analyses across the voxels was much smaller than the number of voxels in gray matter mask, as each voxel may highly dependent on their neighboring voxels because of true physiological correlation and the previous pre-processing steps like spatial registration and smoothing. The the average spatial smoothness of our CBF and FCS maps was FWHMx × FWHMy × FWHMz = 6.26 mm × 7.63 mm × 8.43 mm estimated by “rp_Smoothest” in REST software,^2^ which was much larger than the voxel size (3 mm × 3 mm × 3 mm). Therefore, the spatial correlation would be dominated by the spatial smoothness. Therefore, the *df*_*eff*_ of the CBF-FCS correlation coefficient was calculated based on following formulation:


$$d\,f_{e\,f\,f}=\frac{N}{\left(\text{FWHMx}\times \text{FWHMy}\times \text{FWHMz}\right)/v}-2$$

Where *v* is the voxel size and *N* is the number of voxels (*N* = 67451). Then, we corrected the *p*-value of CBF-FCS correlation coefficient using the *df*_*eff*_. Finally, the CBF-FCS correlation coefficient was obtained from each subject. We evaluated the group differences of CBF-FCS correlation coefficients between the MS patients and HC using Student’s *t*-test. The corrected *p* < 0.05 was considered statistically significant.

#### 2.3.4. Voxel-wise CBF/FCS ratio, CBF, and FCS analyses

The CBF/FCS ratio maps were generated to evaluate the cerebral blood supply per unit of functional connectivity strength for each voxel. The original CBF (ml/100 g/min) and FCS values were used to calculate CBF/FCS ratio and then transformed into z-scores (zCBF/FCS ratio). A general linear model was constructed for zCBF/FCS ratio in SPM8, with both age and sex were included as covariates. Whole-brain voxel-wised independent *t*-test was used for intergroup comparisons and false discovery rate (FDR) method (voxel level *p* < 0.001 and cluster level *p* < 0.05) was used for multiple comparisons correction. In addition, the differences of zCBF and zFCS between two groups at whole-brain voxel level were also explored using independent *t*-tests controlling for age and sex with FDR correction (voxel level *p* < 0.001 and cluster level *p* < 0.05).

#### 2.3.5. ROI-based analysis of CBF and FCS in motor-related brain regions

**FIGURE 1:**
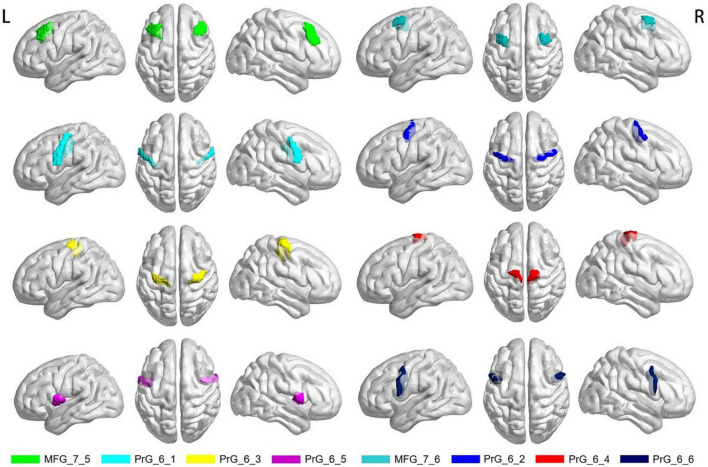
Selected motor-related subregions of interest. MFG, middle frontal gyrus; PrG, precentral gyrus; R, right; L, left.

Previous studies have found changes in neuronal activity in motor-related brain regions in MS patients. The ventrolateral premotor area (ventrolateral area 6), the ventrolateral frontal eye field (ventrolateral area 8) and the primary motor cortex (precentral gyrus) are the key cortical regions for motor function (Mayka et al., 2006). Based on the human Brainnetome Atlas that consists of 246 brain regions (BNA246),^3^ we selected these significant motor-related areas as regions of interest (ROI) to analyze the changes of CBF and FCS in MS patients. The selected motor-related subregions are presented in Table 1 and Figure 1, including bilateral middle frontal gyrus (MFG, MFG_7_5∼6) and bilateral precentral gyrus (PrG, PrG_6_1∼6). As mentioned above, the mean zCBF and zFCS values of each subregion were automatically extracted. The group zCBF- and separately zFCS-values in each subregion were compared, to evaluate differences between MS and HC using Student’s *t*-test. The FDR method was applied for multiple comparisons correction. A value of *q* < 0.05 was considered statistically significant.

**TABLE 1:** Information about motor-related subregions.

ROIs	Anatomical descriptions	lh.MNI coordinates (X, Y, Z)	rh.MNI coordinates (X, Y, Z)
MFG_ 7_5	Ventrolateral area 8	−33, 23, 45	42, 27, 39
MFG_ 7_6	Ventrolateral area 6	−32, 4, 55	34, 8, 54
PrG_ 6_1	Area 4 (head and face region)	−49, −8, 39	55, −2, 33
PrG_ 6_2	Caudal dorsolateral area 6	−32, −9, 58	33, −7, 57
PrG _6_3	Area 4 (upper limb region)	−26, −25, 63	34, −19, 59
PrG _6_4	Area 4 (trunk region)	−13, −20, 73	15, −22, 71
PrG _6_5	Area 4 (tongue and larynx region)	−52, 0, 8	54, 4, 9
PrG _6_6	Caudal ventrolateral area 6	−49, 5, 30	51, 7, 30

ROIs, regions of interest; MFG, middle frontal gyrus; PrG, precentral gyrus; MNI, Montreal Neurological Institute; lh, left hemisphere; rh, right hemisphere.

### 2.4. Clinical data

Statistical analysis was performed on SPSS software (IBM SPSS 21.0). The Shapiro–Wilk test was used to assess the data normality. Independent *t*-tests were used for intergroup comparisons of age and educational level. Differences between groups of different sex were compared using the Chi-square (χ^2^) test. A *p*-value less than 0.05 was considered statistically significant.

## 3. Results

### 3.1. Demographic and clinical characteristics

Data from twenty-five MS patients and thirty age- and sex-matched HC were finally included in the analysis. Demographic characteristics and the scales of disease severity are presented in Table 2. BFMDRS-M scores and BSDI scores were used to assess the severity of dystonia in MS patients. There was no significant difference in sex, age and education between the MS and HC groups (all *P* > 0.05).

**TABLE 2:** Demographics and clinical characteristics.

	MS group (*n* = 25)	HC group (*n* = 30)	*p*-value
Age (years)	57.0 ± 9.5	61.2 ± 7.3	0.064
Gender (M/F)	5/20	10/20	0.269
Education (years)	8.8 ± 4.8	9.0 ± 4.1	0.872
Disease duration (month)	32.1 ± 28.3	NA	NA
BFMDRS-M	7.7 ± 2.7	NA	NA
BSDI	12.9 ± 5.0	NA	NA

Sequential variables are expressed as mean ± standard deviation. MS, Meige syndrome; HC, healthy control; BFMDRS-M, Burke-Fahn-Marsden dystonia rating scale movement; BSDI, blepharospasm disability index; NA, not applicable.

### 3.2. CBF-FCS coupling changes in MS patients

**FIGURE 2:**
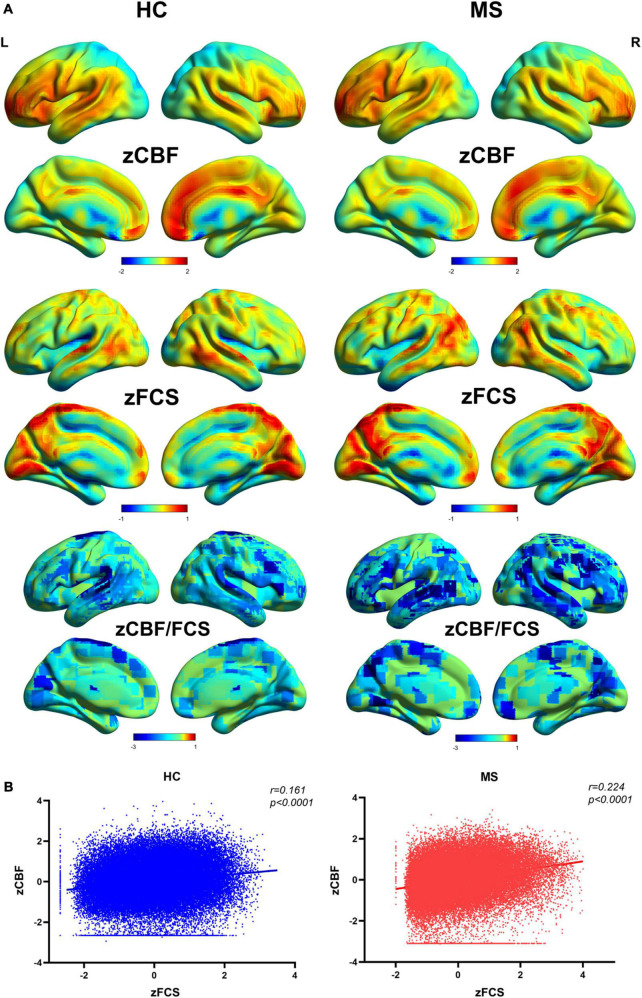
Spatial distribution patterns of FCS, CBF, and CBF/FCS ratio in Meige patients and healthy controls. **(A)** Standardized and averaged FCS, CBF and CBF/FCS ratio maps across each group subjects. **(B)** Scatter plots show a significant spatial correlation (computed over all voxels in the gray matter) between FCS and CBF. We represent data from a healthy participant and a Meige syndrome patient. MS, Meige syndrome; HC, healthy control; CBF, cerebral blood flow; FCS, functional connectivity strength; R, right; L, left.

The distributional patterns of zCBF, zFCS, and zCBF/FCS ratio in MS and HC groups are shown in Figure 2A. The spatial distributions of zCBF, zFCS, and zCBF/FCS ratio were visually similar in MS to those in HC. True for both groups, brain regions – most of which within the most of which were within default mode network (DMN) and salience network (SN) – showed higher CBF and FCS values relative to those in other brain regions. These brain regions included the posterior and anterior cingulate gyrus, precuneus, cingulate cortex, medial and lateral frontal cortex, lateral parietal cortex and lateral temporal cortex. The CBF/FCS ratio was relatively higher in the parts of the medial prefrontal cortex, anterior cingulate cortex, sensorimotor cortex, and thalamus.

**FIGURE 3:**
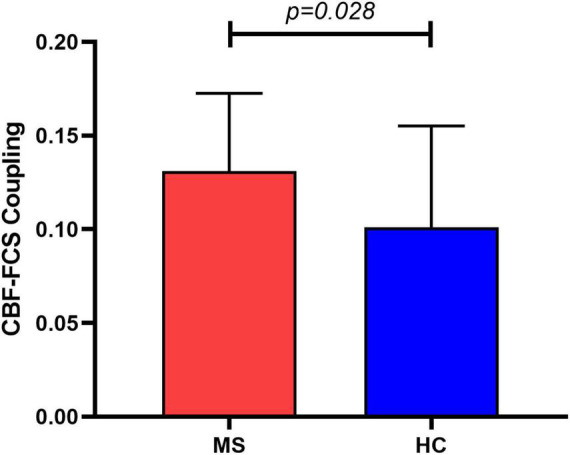
Group comparison of CBF-FCS coupling between Meige syndrome patients and healthy controls. MS patients showed increased CBF-FCS coupling relative to healthy controls. Data are shown as the mean ± std. MS, Meige syndrome; HC, healthy control; std, standard deviation.

The MS patients showed a trend of increased global CBF (MS: 45.8 ± 13.4 ml/100 g/min; HC: 43.2 ± 9.6 ml/100 g/min; *t* = 0.83, *p* = 0.410) and global FCS (MS: 0.059 ± 0.021; HC: 0.051 ± 0.013; *t* = 1.57, *p* = 0.122) in comparison to HC. Evaluating the relationship between CBF and FCS across all voxels in the gray matter showed positive correlation in both MS and HC participants (Figure 2B). Compared with HC, the MS patients had a significantly higher CBF-FCS coupling (*t* = 2.262, *p* = 0.028) (Figure 3).

### 3.3. Whole gray matter analyses of CBF/FCS ratio, CBF, and FCS in MS patients

Cerebral blood flow/FCS ratio, CBF and FCS maps did not show significant differences between MS and HC groups at voxel level following the FDR corrections.

### 3.4. CBF of motor-related brain regions changes in MS patients

**FIGURE 4:**
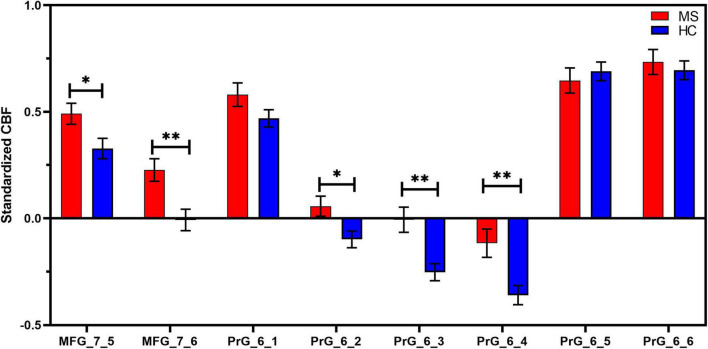
ROI-based CBF intergroup comparisons between Meige syndrome patients and healthy controls. In MFG_7_5, MFG_7_6, PrG_6_2, PrG_6_3, and PrG_6_4, standardized CBF values were significantly higher in MS than in HC (false discovery rate correction, *q* < 0.05). Data are shown as the mean ± SEM. *P < 0.05 (uncorrected), ^**^P < 0.01 (uncorrected). MFG, middle frontal gyrus; PrG, precentral gyrus; MS, Meige syndrome; HC, healthy control; CBF, cerebral blood flow; SEM, standard error of mean.

Compared with HC, MS patients showed increased CBF values in MFG_R_7_5 (MS: 0.49 ± 0.25; HC: 0.33 ± 0.26; *t* = 2.367, *p* = 0.022), MFG_R_7_6 (MS: 0.23 ± 0.26; HC: −0.007 ± 0.28; *t* = 3.199, *p* = 0.002), PrG _6_2 (MS: 0.06 ± 0.24; HC: −0.10 ± 0.21; *t* = 2.578, *p* = 0.013), PrG _6_3 (MS: −0.007 ± 0.30; HC: −0.25 ± 0.22; *t* = 3.544, *p* = 0.001), and PrG _6_4 (MS: −0.12 ± 0.33; HC: −0.36 ± 0.24; *t* = 3.126, *p* = 0.003) following multiple comparisons correction. More detailed information is shown in Figure 4 and Table 3. However, patients with MS exhibited no significant differences in FCS in all selected motor-related ROIs in comparisons to HC.

**TABLE 3:** Results of ROI-based analysis of intergroup differences in standardized CBF.

Brian regions	Standardized CBF value		*t*-value	*p*-value
	**MS group**	**HC group**		
MFG _7_5	0.49 ± 0.25	0.33 ± 0.26	2.367	**0.022**
MFG _7_6	0.23 ± 0.26	−0.007 ± 0.28	3.199	**0.002**
PrG _6_1	0.58 ± 0.28	0.47 ± 0.22	1.659	0.103
PrG _6_2	0.06 ± 0.24	−0.10 ± 0.21	2.578	**0.013**
PrG _6_3	−0.007 ± 0.30	−0.25 ± 0.22	3.544	**0.001**
PrG _6_4	−0.12 ± 0.33	−0.36 ± 0.24	3.126	**0.003**
PrG _6_5	0.65 ± 0.29	0.69 ± 0.24	−0.596	0.554
PrG _6_6	0.73 ± 0.29	0.70 ± 0.24	0.536	0.594

Data are expressed as mean ± standard deviation. *P*-values in the table represent the original value without correction. False discovery rate corrected *q*-values < 0.05 are presented in bold. MS, Meige syndrome; HC, healthy control; MFG, middle frontal gyrus; PrG, precentral gyrus.

## 4. Discussion

In this study, we evaluated the alterations of neurovascular coupling in MS with the combination of resting-state BOLD and ASL techniques. To the best of our knowledge, this is the first study to investigate the relationship between FCS and CBF in MS. Consistent with our hypotheses that neuronal activity or neurovascular coupling may reorganized in MS and CBF may change in motor-related region, our main findings are that: (i) CBF-FCS coupling was higher in MS than that in HC; and (ii) MS patients showed increased CBF value in several motor-related brain regions relative to healthy controls. These findings may provide information to better understand the neural pathophysiological mechanism in Meige syndrome from the perspective of neurovascular coupling.

In agreement with the previous study, we found that CBF and FCS are significantly correlated across the voxels of the gray matter in healthy participants (Liang et al., 2013). We also found significant CBF–FCS correlation in MS patients, however, the coupling strength in MS was higher than that in HC. The normal neurovascular coupling mechanism represents a complete structure of neurons, astrocytes and vascular components (Hawkins and Davis, 2005). According to the neurovascular coupling hypothesis, it can be inferred that changes in CBF are closely related to underlying neuronal activity (Wang et al., 2021). Brain regions with increased neuronal activity have higher metabolic demands and therefore require more perfusion (Venkat et al., 2016). At voxel level, FCS and separately, CBF/FCS ratio demonstrate no significant differences between MS and HC in any local regions. The CBF/FCS ratio depicts the amount of blood supply per unit of connectivity strength which reflects a balance between perfusion and neuro functional activity. Our results demonstrated that tighter neurovascular coupling in MS was constructed. Increased neurovascular coupling may attribute to the changes in the level of neurotransmitters such as dopamine, acetylcholine, norepinephrine and γ-aminobutyric acid (GABA) in MS (Hayashi et al., 1998; Yoshimura et al., 2001; Termsarasab et al., 2016), as these compounds may play a role in regulating neuronal metabolism and vascular responses (Cauli et al., 2004; Tayebati et al., 2011).

Patients with MS have increased CBF values in the primary motor cortex, ventrolateral premotor cortex and ventrolateral frontal eye field, which are mainly involved in abnormal motor executive control and sensorimotor guidance. Compared with isolated blepharospasm patients and normal subjects, patients with Meige syndrome showed abnormal activation of the primary motor cortex and ventral premotor cortex in the oral presentation area (Dresel et al., 2006). Several analyses of resting-state FC in MS patients found that reduced connectivity between the cingulate cortex and the primary sensorimotor/premotor and parietal association cortex, between premotor areas and the primary somatosensory cortex, and between the post-central gyrus and temporoparietal and secondary somatosensory regions (Huang et al., 2017; Jochim et al., 2018). A different study reported increased global FC in the right postcentral gyrus/precentral gyrus/paracentral lobule, right superior frontal gyrus, and left paracentral lobule/supplement motor area (Pan et al., 2021). Positron emission tomography of patients with blepharospasm Meige syndrome showed abnormal activation pattern in the auxiliary motor cortex, which indirectly reflected the abnormal sensory processing phenomenon in the auxiliary motor cortex during the performance of vibration and touch stimulation (Feiwell et al., 1999). These demonstrated that altered brain functional activity in MS patients involved a wide range of brain regions including primary/secondary sensorimotor and auxiliary motor areas, which indirectly indicated a tendency for motor inhibition and sensorimotor integration deficits. However, FCS in this study showed no significant differences between MS and HC in motor-related regions or at the whole-brain voxel level. FCS reflected the functional connectivity of each voxel with all other voxels in the gray matter. One possible explanation of our results is that the reorganization of brain function may need a compensatory supplement of blood to provide more energy and keep the normal functional activity, and our MS patients may be at such a compensatory stage. So, the CBF was elevated, but FCS did not massively change. Another possible explanation is that at the early stage of MS, the brain structures including astrocytes and neurons are not damaged significantly, and therefore no alteration of functional activity takes place. However, the microvasculature alterations may regulate CBF in brain motor regions.

## 5. Limitations

This study also has several limitations. First, the number of subjects is small sample size, which may reduce the statistical effect of group analysis. In future study, we will enlarge the number of participants to further explore the changes of CBF in MS. Second, this study only collected ASL data for one post-label delay, and we will consider collecting ASL data for multiple PLDs in the future. Third, the CBF–FCS correlation was indirect evaluation of neurovascular coupling, and cannot allow us to uncover an accurate pathophysiological mechanism of our findings. Also, we need to use other functional indexes, like ALFF and ReHo, in future study. Fourth, in this study, only the motion-related brain regions that we consider to be meaningful were selected for research, and thus other unselected motor regions can be explored in future researches. Finally, we used a cross-section study design to reveal the alterations in cerebral blood flow, follow-up of longitudinal studies would help us better understand the underlying neural mechanisms of these changes at different disease stages of Meige syndrome.

## 6. Conclusion

In conclusion, patients with Meige syndrome showed altered coupling between resting-state CBF and FCS. Moreover, MS is associated with abnormal CBF increase in motor-related brain areas in MS. Our results provide a new insight into neural mechanism in MS from the perspective of neurovascular coupling and cerebral perfusion.

## Data availability statement

The original contributions presented in this study are included in the article/supplementary material, further inquiries can be directed to the corresponding authors.

## Ethics statement

The studies involving human participants were reviewed and approved by the Ethics Committee of China-Japan Friendship Hospital. The patients/participants provided their written informed consent to participate in this study.

## Author contributions

AY, BL, and KL analyzed and explained the data and drafted and revised the manuscript. GM, HT, SL, and BZ designed the study. GM, HT, SL, BZ, and AS critically revised the manuscript. JL, HY, and AY performed the MRI scanning and evaluated the image quality. PH and KL pursued the literature search and review. All authors approved the final manuscript.

## Footnotes

1
http://www.fil.ion.ucl.ac.uk/spm

2
http://www.restfmri.net/

3
http://atlas.brainnetome.org
